# Case report: Robust response to sintilimab in advanced distal cholangiocarcinoma with PD-L1 expression and DNA damage repair

**DOI:** 10.3389/fphar.2024.1336699

**Published:** 2024-04-10

**Authors:** Wenguang He, Congcong Song, Jiwei Ren, Xiao Ji, Xiuyan Wang, Lixia Liu

**Affiliations:** ^1^ Traditional Chinese Medicine Department, Shanxi Province Cancer Hospital, Shanxi Hospital Affiliated to Cancer Hospital Chinese Academy of Medical Sciences, Cancer Hospital Affiliated to Shanxi Medical University, Taiyuan, China; ^2^ Shenzhen Engineering Center for Translational Medicine of Precision Cancer Immunodiagnosis and Therapy, YuceBio Technology Co., Ltd., Shenzhen, China

**Keywords:** cholangiocarcinoma, sintilimab, PD-L1, DNA damage repair, complete response

## Abstract

Cholangiocarcinoma (CCA) is a highly heterogeneous tumor that occurs in the bile duct epithelium; adenosquamous carcinoma is a rare pathological subtype of CCA. The clinical treatment of patients with metastatic distal CCA poses significant challenges. We report a 53-year-old female diagnosed with a stage III adenosquamous carcinomas of distal CCA. Metastasis occurred 4 months postoperatively and she was diagnosed with stage IV disease. The patient was treated with Gemcitabine + Oxaliplatin (GEMOX) and Capecitabine + Oxaliplatin (CAPEOX), followed by sintilimab monotherapy. After two cycles of treatment, the patient achieved partial response (PR) and the lesion continued to shrink. After 37 months of follow-up, the patient’s liver metastasis had almost completely disappeared, and complete response (CR) was achieved. Moreover, she had more than 46 months of disease progression-free survival (PFS). Immunohistochemical testing showed high expression of PD-L1, and next-generation sequencing revealed the presence of mutations in DNA damage repair (DDR) pathway genes. To the best of our knowledge, this is the first reported case of the successful treatment of metastatic distal adenosquamous CCA with sintilimab alone. Remarkably, patients of CCA with high PD-L1 expression and DDR pathway gene mutations may benefit from sintilimab treatment.

## Introduction

Cholangiocarcinoma (CCA) is a lethal tumor of the bile duct epithelium. CCAs can be divided into three subtypes based on the anatomical location of their origin, intrahepatic cholangiocarcinoma (iCCA), perihilar cholangiocarcinoma (pCCA), and distal cholangiocarcinoma (dCCA). pCCA and dCCA can also be collectively referred to as “extrahepatic” cholangiocarcinoma (eCCA). CCA has a poor prognosis due to its late diagnosis and limited treatment options; it is estimated that the 5-year survival rate is 7%–20% (Banales et al., 2020).

Chemotherapy is the most common first-line treatment for patients with metastatic CCA; however, patients with short progression-free survival (PFS) and poor quality of life, and do not have good treatment options when disease progression. Adenocarcinoma is the most common histological subtype of CCA, accounting for more than 90% of eCCA, while adenosquamous cell carcinoma is extremely rare, accounting for only 2%–5% of the eCCA cases (Hong et al., 2021). The malignancy of the adenosquamous subtype is higher than that of adenocarcinoma, and thus the overall survival period is shorter (Sapuppo et al., 2023). Therefore, the treatment of adenosquamous carcinoma of dCCA is challenging.

Herein, we report a case of a patient with multiple metastatic adenosquamous carcinomas of the dCCA. After third-line treatment with sintilimab, the patient achieved complete response (CR) and a good quality of life. PFS exceeded 46 months, and encouraging clinical results were achieved. The high expression of PD-L1 in this patient and the presence of mutations in the DNA damage repair (DDR) pathway genes suggest that these can be used as markers to guide sintilimab treatment.

## Case presentation

**FIGURE 1:**
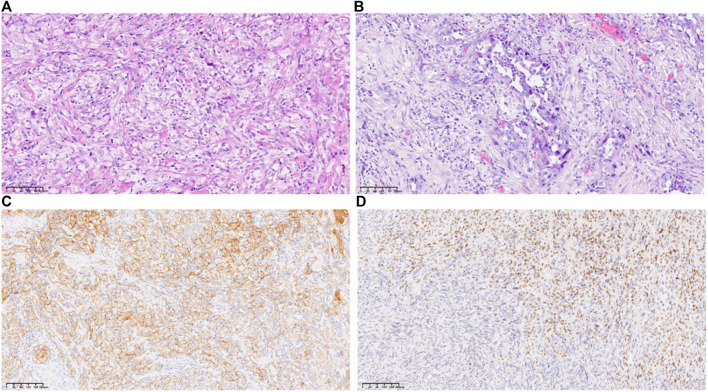
Histology of the tumor tissue **(A, B)**, hematoxylin and eosin, original magnification ×200; **(C)**, immunohistochemistry of AE1/AE3, original magnification ×100; **(D)**, immunohistochemistry of P40, original magnification ×100.

A 53-year-old female was admitted to Jincheng Hospital on 8 July 2018, due to “upper abdominal fullness and discomfort, together with yellow skin staining for 1 week.” On 16 July 2018, abdominal magnetic resonance imaging (MRI) revealed 1) irregular thickening of the common bile duct and the upper segment of the pancreas, considering common bile duct cancer with biliary obstruction, and 2) multiple enlarged lymph nodes in the hilar area of the liver. On 16 August 2018, radical resection of the common bile duct cancer and combined pancreaticoduodenectomy were performed under general anesthesia, at the Shanxi Provincial Cancer Hospital, and R0 resection was achieved. The partial pancreaticoduodenal cholecystectomy specimens were subjected to postoperative pathological examination. The common bile duct: poorly differentiated carcinoma, combined with immunohistochemical results: AE1/AE3 (+), CAM5.2 (+), CGA (−), Syn (−), Ki67 (approximately 60%+), P40 (partly+), P63 (partly+), consistent with adenosquamous cell carcinoma (approximately 30% squamous cell carcinoma), with a tumor size of 3 cm * 2 cm * 1 cm, infiltrating the common bile duct to the outside of the serosa, adjacent to pancreatic tissue. No neuro-vascular involvement was seen (Figure 1). The gallbladder: chronic inflammation of the mucosa with cholesterol polyps. The gastric incision margin, intestinal incision margin, and omentum: no carcinoma was found. No metastatic cancer was found in the lymph nodes: 0/2 in the greater curvature, 0/2 in the mesentery, 0/1 around the pancreas, and 0/1 in the 12b group. Postoperative diagnosis revealed adenosquamous carcinoma of the lower part of the common bile duct, TNM stage III, pT3N0M0.

On 17 December 2018, an abdominal computed tomography was done at Shanxi Provincial Cancer Hospital. It showed that after the operation for bile duct adenosquamous carcinoma, multiple nodules and masses near the stoma of the remnant stomach, mesentery, and retroperitoneum were considered to have metastasized. The nodules in the right anterior segment of the liver were considered to have metastasized, and intrahepatic bile duct had gas accumulation. The diagnosis at admission revealed adenosquamous carcinoma of the lower part of the common bile duct (TNM stage IV, TxNxM1), performance status (PS) score was 3. From 2 January 2019, Gemcitabine + Oxaliplatin (GEMOX) chemotherapy was started, and a total of two cycles was administered. From 2 April 2019, CT was performed and indicated the progress of the disease. Thereafter, a Capecitabine + Oxaliplatin (CAPEOX) chemotherapy regimen was administered for one cycle.

**FIGURE 2:**
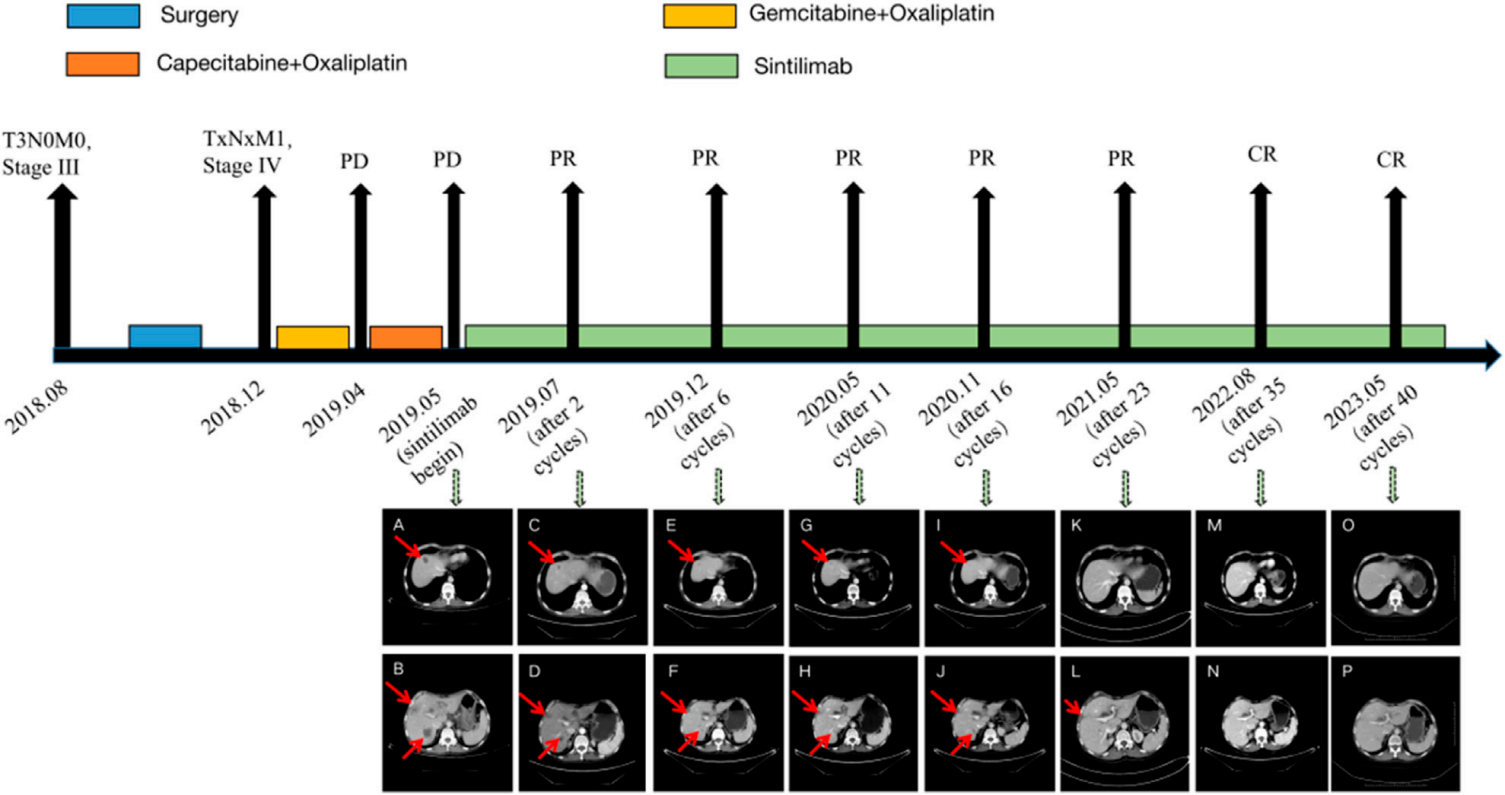
The case timeline and CT images before and after treatment with sintilimab. **(A,B)**, sintilimab begin; **(C,D)**, 2 cycles after sintilimab treatment; **(E,F)**, 6 cycles after sintilimab treatment; **(G,H)**, 11 cycles after sintilimab treatment; **(I,J)**, 16 cycles after sintilimab treatment; **(K,L)**, 23 cycles after sintilimab treatment; **(M,N)**, 35 cycles after sintilimab treatment; **(O,P)**, 40 cycles after sintilimab treatment.

On 20 May 2019, the patient was readmitted because of upper abdominal pain, nausea, vomiting, and inability to eat. PS score was 4, numerical rating scale (NRS) score was 5. On 23 May 2019, a CT scan of the chest and upper abdomen showed that the mass near the anastomosis, mesentery, and retroperitoneum was now larger than that in the old film from 2 April 2019. The new liver parenchyma mass and nodule occupied the space, resulting in intrahepatic bile duct dilatation (Figures 2A,B), and disease progression. Subsequently, 200 mg sintilimab was administered on 27 May 2019, and 1 July 2019, for two cycles. On 22 July 2019, the patient was admitted again, and symptoms, such as upper abdominal pain, nausea, vomiting, and inability to eat were completely alleviated. PS score was 1, NRS score was 0. On 23 July 2019, the CT scan of the neck, chest, abdomen, and pelvis showed that the mesentery and retroperitoneal lesions were reduced in size, and the liver parenchymal lesions were reduced compared with those on the CT scan from 23 May 2019^−ΔΔCT^ scan (Figures 2C,D). According to RECIST 1.1, the efficacy was evaluated as a partial response (PR), NRS score was 0. Subsequently, the patient continued on sintilimab 200 mg Q3W injection. After six cycles of treatment, CT evaluation was conducted again on 13 December 2019. Compared with 23 July 2019, the liver nodules had disappeared; however, the S8 segment of the liver still had nodules, and therefore, regular observation was recommended. Furthermore, intrahepatic bile duct dilatation was slightly relieved; hilar and retroperitoneal lymph nodes had significantly decreased in size and partially disappeared (Figures 2E,F), and thus the efficacy was evaluated as PR. On 20 May 2020, CT showed that the lesions near the anastomosis, mesentery, and retroperitoneum were smaller than before, and the lesions of the liver parenchyma were smaller (Figures 2G,H). On 22 November 2020, CT examination showed that the nodules in the S8 segment of the liver had shrunk compared with those in previous images, and no significant changes were observed in the remaining segments (Figures 2I,J). On 27 May 2021, CT showed that the low-density shadows in the S8 segment of the liver were slightly smaller than before, and no significant changes were observed in the rest (Figures 2K,L). On 25 August 2022, CT showed that the liver metastases almost completely disappeared, and the efficacy evaluation was of complete response (CR) (Figures 2M,N). At the last follow-up on 22 May 2023, CT showed that the metastatic liver lesion had almost completely disappeared, and the efficacy evaluation showed a CR (Figures 2O,P). The patient’s condition continued to improve during the single-drug treatment period. By 22 May 2023, the patient had received 40 cycles of sintilimab injections, and the PFS was more than 46 months. Currently, the patient is continuing treatment with sintilimab. During the treatment period, the patient was generally well, without any complaints of discomfort or adverse events related to the medication, and the NRS score is 0 at each follow-up. Regular examination showed no abnormalities in routine blood, liver and kidney function, blood sugar, thyroid function, and other indicators (Supplementary Figure S1).

**FIGURE 3:**
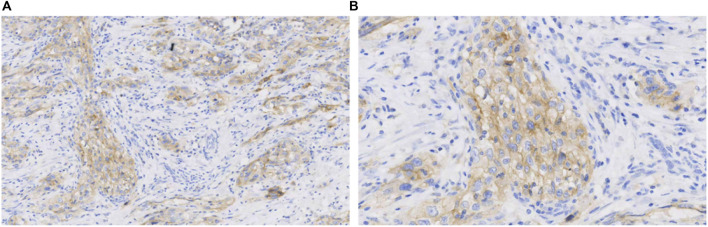
Immunohistochemistry of PD-L1 (22C3) **(A)**, original magnification ×100; **(B)**, original magnification ×400.

This retrospective study was conducted using postoperative tissue samples collected in 2018. Immunohistochemistry revealed PD-L1 (22C3; Tumor Proportion Score (TPS) = 60%, Combined Positive Score (CPS) = 65), MLH1 (+), PMS2 (+), MSH2 (+), and MSH6 (+). Immunohistochemistry showed that PD-L1 was highly expressed (Figure 3), and the mismatch repair (MMR) status was of proficient mismatch repair (pMMR).

Meanwhile, tissue samples were sequenced using cancer-related 1012 gene panel (YuceOne Pro) for next-generation sequencing (NGS), with tumor mutation burden (TMB) = 8.03 Muts/Mb. Moreover, there were mutations in genes involved in the DDR pathway, including somatic mutations *ATM* (p.Arg250Ter, mutation abundance 15.93%), *FANCC* (p.Leu234Val, mutation abundance 15.91%), as well as germline mutations *FANCA* (p.Arg1409Trp, heterozygous), *FANCD2* (p.Leu1325Met, heterozygous) (Supplementary Figure S2). Studies have shown that the DDR pathway affects the immune response of patients. NGS did not reveal any other valuable mutation information. This case suggests that patients with advanced CCA and high PD-L1 expression and mutations in the DDR pathway may benefit from sintilimab treatment.

## Discussion

Cholangiocarcinomas are a group of highly heterogeneous malignant biliary tumors that can occur at any point in the bile duct tree, its incidence has been increasing worldwide (Banales et al., 2020). Complete surgical resection is the treatment method that provides the best chance of a cure for CCA patients. However, it is prone to recurrence after surgery, with a 1-year disease-free survival (DFS) of only 50%–60% (Hu et al., 2020; Rao et al., 2022). According to the results of the phase 3 study of ABC-02, combination chemotherapy with gemcitabine and cisplatin was established as the standard first-line treatment for advanced biliary cancer. The median PFS of gemcitabine combined with cisplatin was 8.0 months (95% CI, 6.6–8.6), and the total survival period was 11.7 months (95% CI, 9.5–14.3) (Valle et al., 2010). There are currently no effective clinical treatment strategies for advanced CCA. Compared with patients having intrahepatic CCA, patients with extrahepatic CCA have a lower molecular spectrum analysis rate, and their use rate of targeted therapy and clinical trial registration rates are also very low. Therefore, there is an urgent need for new effective targeted therapies and more extensive clinical trials (Spencer et al., 2023).

Sintilimab is an engineered PD-1 inhibitor that blocks the interactions between PD-1 and its ligands. It shows better PD-1 binding affinity *in vitro* than nivolumab and pembrolizumab, and superior PD-1 occupancy and antitumor effects in humanized mouse models (Wang et al., 2019). Sintilimab exhibited efficacy in both adenocarcinoma and squamous cell carcinoma pathological types, such as esophageal squamous cell carcinoma (Xu et al., 2022a), squamous non-small cell lung cancer (NSCLC) (Shi et al., 2022), non-squamous NSCLC (Zhang et al., 2022), and gastric adenocarcinoma (Jiang et al., 2022). At present, sintilimab has been included in medical insurance in China, which can greatly reduce the economic burden on patients. A multicenter trial evaluated the efficacy and safety of sintilimab combined with lenvatinib in the second-line treatment of advanced intrahepatic CCA in the real world; 41 patients with advanced iCCA participated in this multicenter observational study, with a median time to progression (TTP) of 6.6 months (95% CI, 4.9–8.3). The objective response rate (ORR) and disease control rate (DCR) was 46.3% and 70.3%, respectively. The median TTP of patients with PD-L1 TPS ≥10% significantly improved to 16.9 months (95% CI, 7.5–26.3), compared with 4.1 months (95% CI, 1.8–6.4), *p* = 0.001 (Ding et al., 2022). In a case report of a patient with advanced iCCA receiving sintilimab combined with gemcitabine and cisplatin as first-line treatment, and sintilimab combined with capecitabine as maintenance treatment, the curative effect was significant, and the PFS exceeded 16 months (Wang et al., 2021). The clinical benefits of PR were observed in a patient with metastatic intrahepatic CCA who failed to receive first- and second-line chemotherapy, but received a third-line treatment with sintilimab combined with tegafur-gimeracil-oteracil potassium capsules (S-1); however, the author only followed up for 1 month (Liu et al., 2020). A patient with recurrent perihilar CCA received first-line treatment of gemcitabine and cisplatin combined with radiofrequency ablation. After the disease progressed, the patient was treated with sintilimab combined with lenvatinib and S-1, and after 14 cycles of combined treatment, the patient achieved CR, and a relapse free survival period exceeding 8 months (Liu et al., 2023). A patient with intrahepatic CCA metastasis was included in the Phase I clinical study of sintilimab due to the failure of first-line chemotherapy. After three cycles of treatment, the patient had mild adverse events and achieved CR, the patient experienced recurrence after 12 cycles of treatment with sintilimab. Genetic testing showed low MSI, and a TMB = 15.1 mut/Mb (Zhang et al., 2020). To the best of our knowledge, the only reports of sintilimab in CCA are on intrahepatic CCA; only one case reports CCA treatment with single drug sintilimab, and the rest report combined sintilimab treatments. Although immunotherapy combination therapy (such as gemcitabine + cisplatin + duvalizumab) has been recommended as first-line treatment according to the National Comprehensive Cancer Network (NCCN) and Chinese Society of Clinical Oncology (CSCO) guidelines, the PFS is limited, with a median PFS of no more than 13 months (Oh et al., 2022). The PFS in this case has exceeded 46 months. We are the first to report a patient with distal CCA who received significant clinical benefits from sintilimab monotherapy for more than 46 months without obvious adverse events. This provides a valuable reference for the clinical treatment of different CCA subtypes.

The detection of molecular markers may be necessary to ensure benefit from immunotherapy. The expression of PD-L1 is often considered to be related to the response to immunotherapy (Doroshow et al., 2021), such as in NSCLC (Herbst et al., 2020; Reck et al., 2021), cervical cancer (Xu et al., 2022b), triple-negative breast cancer (Schmid et al., 2018), head and neck squamous cell carcinoma (Harrington et al., 2023), CAA (Ding et al., 2022) and so on. In clinical studies of many cancer types, PD-L1 positive individuals have achieved significant survival benefits with the use of sintilimab. The real-world study of sintilimab combined with lenvatinib in the treatment of CAA showed that the median TTP of patients with PD-L1 TPS ≥10% was significantly improved (Ding et al., 2022). In a neoadjuvant clinical study of sintilimab as a single drug for NSCLC, patients with PD-L1 ≥ 1% had significantly longer DFS than those with PD-L1<1% (HR, 0.275; 95% CI, 0.078–0.976, *p* = 0.0325) (Zhang et al., 2022). Among patients with unresectable locally advanced or metastatic gastric and gastroesophageal junction adenocarcinoma treated with first-line chemotherapy, sintilimab significantly improved overall survival for all patients and for patients with a CPS of 5 or more compared with placebo (Xu et al., 2023). Sintilimab plus anlotinib as second-line or later therapy is efficacious and safe for patients with advanced cervical cancer with PD-L1-positive (CPS ≥1) (Xu et al., 2022a). Therefore, we speculate that the high expression of PD-L1 in this patient is likely to respond to the treatment with sintilimab. The DDR is related to the immune response of patients. Cancer cells with DDR defects can be recognized by the immune system as foreign cells, and excessive DNA damage can cause an immune response via cell death signals (Jiang et al., 2021). Patients with mutations in the DDR pathway have significantly better efficacy with respect to immunotherapy, which has been reported in various cancers, such as lung cancer (Zhou et al., 2022), urothelial carcinoma (van Dijk et al., 2020), colorectal cancer (Song et al., 2021), and others. The patient reported in this case had high expression of PD-L1 and changes in the DDR pathway genes, indicating that the patient could not only effectively preserve T-cell anti-tumor activity, but also that the immune system could recognize more tumor cells for killing. Our case suggests that patients with advanced CCA who have high expression of PD-L1 and mutations in the DDR pathway can benefit from treatment with sintilimab. As we know, patients with metastatic cholangiocarcinoma who have MSI-H or dMMR can be treated with pembrolizumab (Marabelle et al., 2020; Maio et al., 2022), but this patient has pMMR, indicating that clinical researchers can use other molecular biomarkers, such as PD-L1 and DDR, to indicate the immunotherapy benefits of cholangiocarcinoma patients even in pMMR status. The detection of biomarkers will help provide more accurate immunomonotherapy for patients, reducing their adverse events and the economic burden. However, larger clinical cohorts are still needed for validation.

## Conclusion

This is the first report of a patient with advanced adenosquamous carcinoma of the dCCA who failed to receive second-line chemotherapy, in whom third-line sintilimab monotherapy achieved significant clinical benefits. The patient’s PFS was >46 months without obvious adverse events. Tissue immunohistochemistry showed that PD-L1 was highly expressed, and NGS test showed a mutation in the DDR pathway gene, suggesting that these may be used as an immune markers for patients to respond to sintilimab. However, a larger clinical cohort study is needed to study the effectiveness of sintilimab in patients with high PD-L1 expression and DDR mutation CAA to guide patients to precise immunotherapy.

## Figures and Tables

**Table:** 

**ALT**	Alanine aminotransferase
**AST**	Aspartate aminotransferase
**CAPEOX**	Capecitabine + Oxaliplatin
**CCA**	Cholangiocarcinoma
**CPS**	Combined positive score
**CR**	Complete response
**CSCO**	Chinese Society of Clinical Oncology
**DBIL**	Direct bilirubin
**dCCA**	Distal cholangiocarcinoma
**DCR**	Disease control rate
**DDR**	DNA damage repair
**DFS**	Disease-free survival
**eCCA**	Extrahepatic cholangiocarcinoma
**GGT**	Gamma-glutamyltransferase
**GEMOX**	Gemcitabine + Oxaliplatin
**IBIL**	Indirect bilirubin
**iCCA**	Intrahepatic cholangiocarcinoma
**LDH**	Lactate dehydrogenase
**MMR**	Mismatch repair
**MRI**	Magnetic resonance imaging
**NCCN**	National Comprehensive Cancer Network
**NGS**	Next-generation sequencing
**NRS**	Numerical rating scale
**NSCLC**	Non-small cell lung cancer
**ORR**	Objective response rate
**pCCA**	Perihilar cholangiocarcinoma
**PFS**	Progression-free survival
**pMMR**	Proficient mismatch repair
**PR**	Partial response
**PS**	Performance status
**S-1**	Tegafur-gimeracil-oteracil potassium capsules
**TBIL**	Total bilirubin
**TMB**	Tumor mutation burden
**TPS**	Tumor proportion score
**TTP**	Time to progression

## Data Availability

The original contributions presented in the study are included in the article/Supplementary Material, further inquiries can be directed to the corresponding author.

## Supplementary material

Supplementary Figure S1 Liver biomarkers of this patient ALT, alanine aminotransferase(reference range: 7-40 u/L); AST, aspartate aminotransferase(reference range: 13-35u/L); LDH, lactate dehydrogenase(reference range: 120-250 u/L); GGT, gamma-glutamyltransferase(reference range: 7-45 u/L); TBIL, total bilirubin(reference range: ≤23.0 umol/L); DBIL, direct bilirubin(reference range: ≤4.0 umol/L); IBIL, indirect bilirubin(reference range: ≤19.0 umol/L).

Supplementary Figure S2 Gene mutation information of NGS.
