# Supplementary figures and images for: Case report: Robust response to sintilimab in advanced distal cholangiocarcinoma with PD-L1 expression and DNA damage repair
Source: Front Pharmacol. 2024 Apr 10;15:1336699. doi: 10.3389/fphar.2024.1336699 (PMC11039799; doi:10.3389/fphar.2024.1336699)

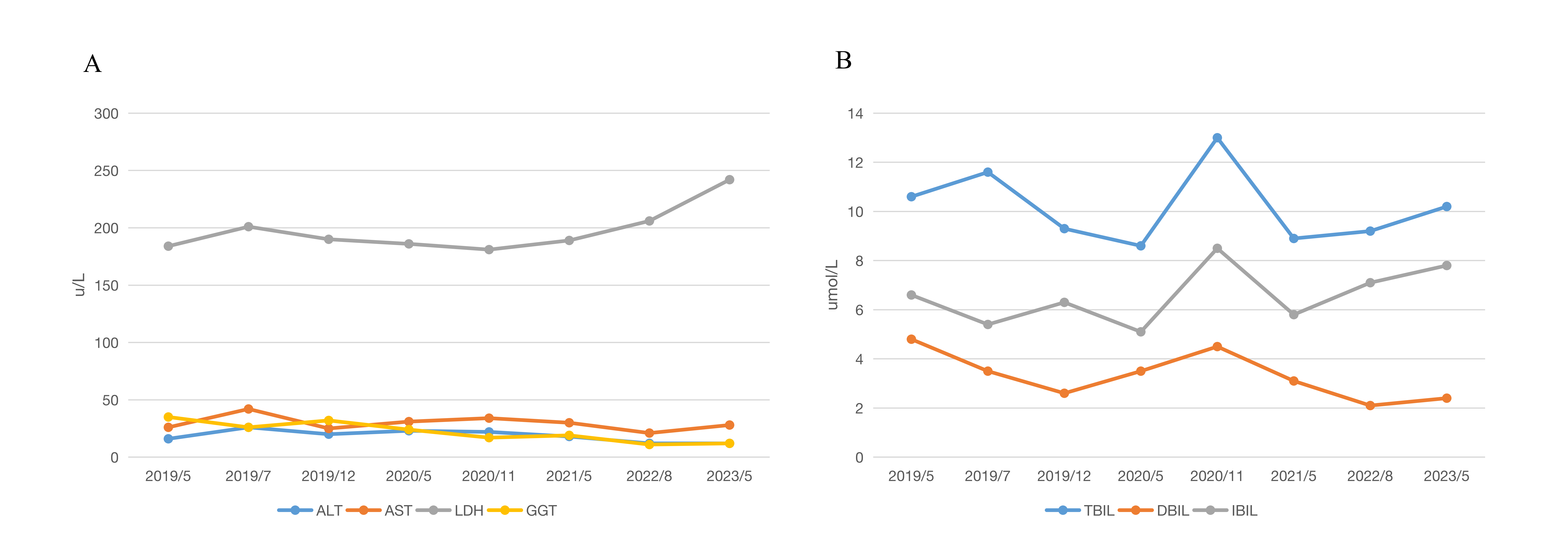

Supplement: Supplementary file 1 [file Image1.JPEG]

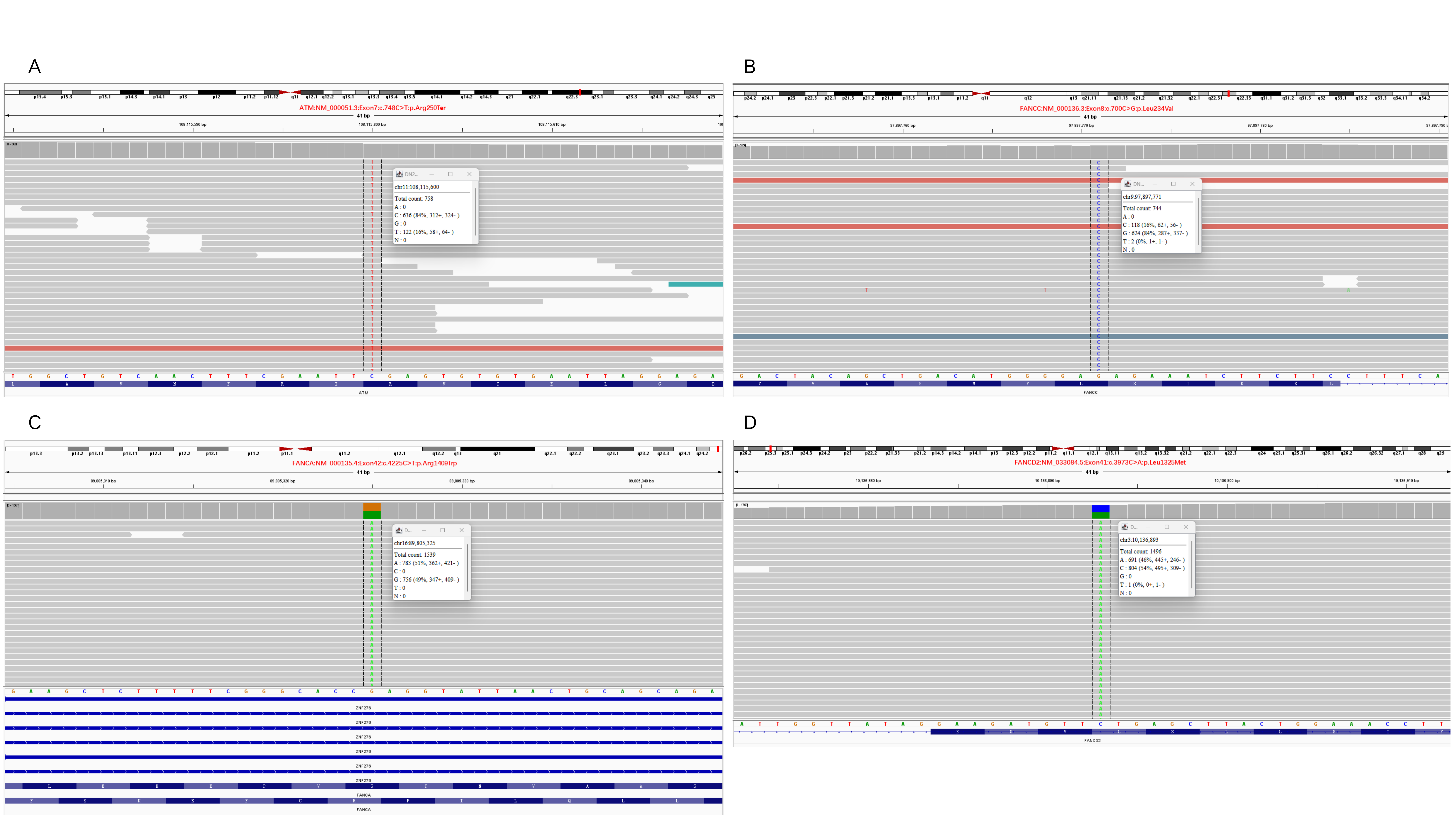

Supplement: Supplementary file 2 [file Image2.JPEG]
